# The effectiveness of the diversion of patients from an emergency department waiting room to a virtual medical consultation

**DOI:** 10.1093/oodh/oqag018

**Published:** 2026-07-11

**Authors:** Jennie Hutton, Clare Matheson, Suzanne Miller, Jason Talevski, Rebecca L Jessup, Adam I Semciw, James H Boyd, Hayley Gray, Abbey Gwyther-Jones, Vivienne King, Joanna Lawrence, Loren Sher

**Affiliations:** Victorian Virtual Emergency Department, Northern Health, 185 Cooper St Epping VIC 3076, Victoria, Australia; School of Psychology and Public Health, La Trobe University, Plenty Rd, Bundoora, VIC 3086, Victoria, Australia; University of Melbourne, Grattan Street, Parkville, Victoria 3010, Australia; Victorian Virtual Emergency Department, Northern Health, 185 Cooper St Epping VIC 3076, Victoria, Australia; Victorian Virtual Emergency Department, Northern Health, 185 Cooper St Epping VIC 3076, Victoria, Australia; University of Melbourne, Grattan Street, Parkville, Victoria 3010, Australia; School of Public Health and Preventative Medicine, Monash University, 553 St Kilda Rd, Melbourne, VIC 3004, Australia; Victorian Centre for Virtual Health Research, Northern Health, Melbourne, Victoria, Australia; School of Allied Health, Human Services and Sport, La Trobe University, Melbourne, Victoria, Australia; Victorian Centre for Virtual Health Research, Northern Health, Melbourne, Victoria, Australia; School of Allied Health, Human Services and Sport, La Trobe University, Melbourne, Victoria, Australia; Victorian Virtual Emergency Department, Northern Health, 185 Cooper St Epping VIC 3076, Victoria, Australia; School of Psychology and Public Health, La Trobe University, Plenty Rd, Bundoora, VIC 3086, Victoria, Australia; Victorian Virtual Emergency Department, Northern Health, 185 Cooper St Epping VIC 3076, Victoria, Australia; Victorian Virtual Emergency Department, Northern Health, 185 Cooper St Epping VIC 3076, Victoria, Australia; Victorian Virtual Emergency Department, Northern Health, 185 Cooper St Epping VIC 3076, Victoria, Australia; Victorian Virtual Emergency Department, Northern Health, 185 Cooper St Epping VIC 3076, Victoria, Australia; School of Psychology and Public Health, La Trobe University, Plenty Rd, Bundoora, VIC 3086, Victoria, Australia; University of Melbourne, Grattan Street, Parkville, Victoria 3010, Australia; Victorian Virtual Emergency Department, Northern Health, 185 Cooper St Epping VIC 3076, Victoria, Australia; School of Psychology and Public Health, La Trobe University, Plenty Rd, Bundoora, VIC 3086, Victoria, Australia; University of Melbourne, Grattan Street, Parkville, Victoria 3010, Australia

**Keywords:** telemedicine, triage, emergency service hospital, waiting room, health services accessibility, emergency medicine

## Abstract

The increase in number of presentations to Emergency Departments (EDs) has continued to contribute to poor health outcomes worldwide. The diversion of suitable patients from the waiting room to receive a virtual medical consultation potentially addresses this problem. This retrospective study was undertaken at a metropolitan ED utilizing the public state-wide Victorian Virtual Emergency Department (VVED) service. Nonurgent patients were identified and offered services in a soundproof room adjacent to the waiting room. The proportions of successful diversions from the physical ED and subsequent presentations in the following 7 days were reported. A review of all representations to the hospital was undertaken via a modified Institute of Healthcare Improvement framework. A total of 339 patients were treated via this pathway, resulting in 258 (76%) of patients being diverted from the ED. Eighty-one (24%) participants were advised to visit the ED as planned. A total of 206 participants (61%) had no further contact with the VVED or the ED. A total of 20 (6%) patients undertook a subsequent VVED consultation, and 30 (9%) presented to the ED in ˂7 days and two were directly admitted to a short-stay unit. Twelve patients had hospital admissions of ˂7 days, and no deaths were reported. In the case reviews, three patients were identified as having an adverse event resulting in temporary harm. Virtual consultation services can be integrated into the ED waiting room, resulting in the successful diversion of patients with a small number of adverse events.

## Background

The COVID-19 pandemic placed additional demands on emergency departments (EDs) already overburdened from continuous growth over a number of years (Berchet 2015; Peterson et al. 2019). With few health services able to adequately expand to meet this demand, EDs are overcrowded and have long wait times. Ambulance ramping has become the norm, and all these effects have detrimental impacts on patients and health providers, with increased rates of patient morbidity and mortality and higher operating costs for facilities (Richardson 2006; Sprivulis et al. 2006; Sun et al. 2013; Morley et al. 2018). Implementing early interventions in a patient’s journey and using senior doctors, such as stationing physicians at the triage stage of an ED presentation, has been found to be highly effective in reducing ED crowding (Bittencourt et al. 2020; Youssef et al. 2024).

During the COVID-19 pandemic, telehealth has grown from an alternative mode of care to an essential part of modern healthcare service delivery (Shaver 2022). Virtual healthcare interventions can reduce the demand for crowded EDs and protect vulnerable patients and healthcare workers from exposure to infectious diseases (Rademacher et al. 2019; Houze-Cerfon et al. 2019). Research on telehealth has increased in the last three years, with evidence showing that it can improve patient outcomes across multiple settings (Snoswell et al. 2021; Grygorian et al. 2024). There are reports of the success of different models of telehealth, whereby the telehealth physician triages low-acuity patients from in-person ED waiting rooms, and ongoing treatment occurs at the ED. This approach has been described previously as ‘telescreening’ (Tolia et al. 2016; Rademacher et al. 2019; Friedman et al. 2021); however, few reports exist on the safety of telehealth consultations undertaken for urgent conditions, whereby the aim is for treatment to be completed by a virtual consultation from the waiting room (Sharma et al. 2017; Hsu et al. 2020). Two previous studies in adult EDs reported that patients were very satisfied with the service; in addition, one of these studies reported that all of the patients perceived the intervention to be safe (Hsu et al. 2020; Osmanlliu et al. 2022). In addition, it has been found to be acceptable for patients over 65 years of age, and patients’ time in the ED is reduced by at least 90 min (Sharma et al. 2017; Greenwald et al. 2019). Notably, there were similar representation rates to those seen in person (Hsu et al. 2020). In a pediatric study of this model of care, 12.3% of all ED patients presented could be diverted (Osmanlliu et al. 2022).

To our knowledge, there are no reports on the use of a nurse navigator to identify and support the diversion of patients from physical triage to a virtual emergency review with the aim of completing care without further ED investigations.

This report aims to assess the effectiveness of a pathway to identify and divert patients from an ED waiting room to a virtual consultation by a medical practitioner. A further outcome was to describe the safety of this pathway as identified by a clinical audit of patients who presented to the physical ED and documented these adverse events. A secondary outcome was to compare the rates of effectiveness of the pathway when a dedicated nurse navigator was employed to the period where the role was incorporated into that of the triage nurse.

## Methods

### Study design and setting

This study was implemented for a 5-month period from March to July 2023 and was evaluated retrospectively. The main hospital has over 550 acute beds and provides secondary care to the outer northern suburbs of Melbourne. Care provided by the hospital includes acute, subacute, mental health, specialist, community, maternity, home-based services and a large pediatric service. The hospital’s physical ED is the busiest in the state, with ~300 presentations per day, and the second busiest in Australia.

The Victorian Virtual Emergency Department (VVED) is a public, free-to-access, 24-h, 7-day-a-week health care service established in October 2020 as a way of diverting low-acuity patients from hospital EDs during the COVID-19 pandemic (Talevski et al. 2024). The VVED provides patients with several pathways for telehealth consultations to assess and facilitate either complete or streamlined ongoing care. These pathways include both self-referrals and referrals where patients are assisted by a healthcare provider (e.g. ambulance paramedics, nurses at aged care facilities, and nurses in urgent care centers). The VVED has seen ˃200,000 patients across Victoria, Australia, from July 2023 to June 2024 and averages >750 presentations per day. While the VVED service was launched statewide in February 2022, most patients in the early months resided in the Northern Health catchment, and this study focused on Northern Health’s in-person ED-to-VVED referral pathway.

### Participants

Patients aged ≥18 years or pediatric patients with a parent/guardian who presented to the Northern Hospital ED during the study period and who were deemed suitable for a telehealth appointment following triage were included (Supplementary Material 1). Participants were included only if they resided in the Northern Health catchment area as identified by the postcode of their address at registration.

### Intervention

**Figure 1:**
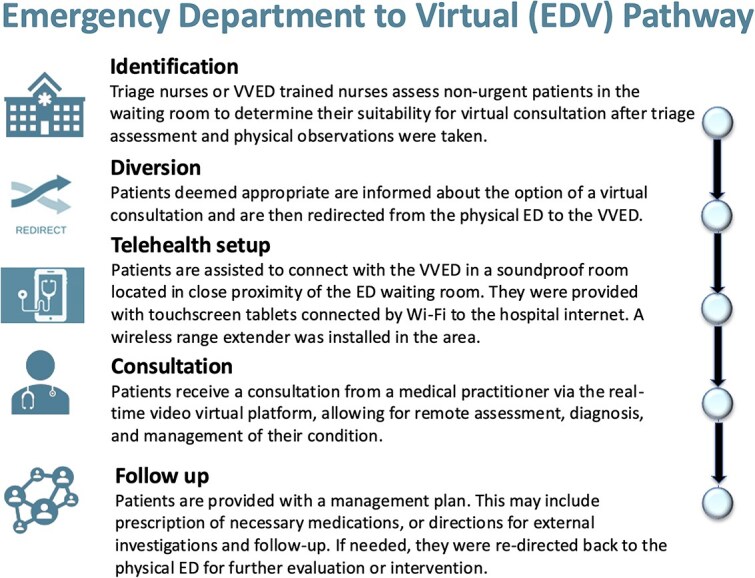
Emergency department to virtual (EDV) pathway.

The ED-to-virtual (EDV) pathway is a novel model of care established in 2023 whereby patients presenting to a physical ED are assessed as appropriate for a virtual consultation and diverted from the ED waiting room to a purpose-built virtual enabled soundproof space (VVED room) to undertake a virtual ED consultation with a medical practitioner (Supplementary Material 2). The EDV pathway involves several key steps, as illustrated in Fig. 1.

For the first 9-week period, there was no navigator nurse employed; from the second 11-week period, there was a dedicated nurse navigator employed from 7:00 am until 9:30 pm seven days a week. These hours were chosen because ~75% of patients present within these hours (~225 per day). During the first period, the triage nurse undertook the role of identifying and referring suitable patients to the VVED in addition to their normal duties. The instructions for the service were detailed in the dedicated room adjacent to the waiting area. During the second period, a dedicated nurse navigator was employed to identify patients from the triage notes and refer patients in the waiting room to attend a VVED consultation in the adjacent room. This nurse navigator also provided assistance in connecting to the service. The nurse navigators were all experienced in working at the VVED service and had no other roles to perform.

At the time of the study, 234 clinicians were employed at Northern Health in the VVED. This consisted of 56 emergency physicians, 105 general practitioners (GPs), 25 pediatricians, 45 nurse practitioners, and three career medical officers. During the period of the intervention, it was estimated that the participants were seen by GPs ~50% of the time and could receive further advice from emergency physicians whom they were working with concurrently.

### Outcomes

The primary outcome of this study was the number and proportion of patients diverted from the physical ED to the VVED, who required no further consultation, and the number of patients who attended the physical ED within 72 h and seven days following the consultation. A secondary outcome was to document the adverse outcomes of participants who had consulted with the VVED and had used the EDV pathway. A further outcome was to compare the number and proportion of patients successfully diverted from the ED when a dedicated VVED nurse navigator was employed compared with when patients were identified and referred by triage nurses as part of their standard role.

### Data sources and analysis

To address the primary outcomes, routinely collected medical information was obtained from the Northern Health administration database and the electronic medical records system. This included demographic and health condition details in addition to all-cause re-presentations and admissions within the seven days following VVED consultation. In addition, data on presentations and admissions to the hospital in the seven days prior to the index ED presentation were collected.

To address the secondary outcomes, clinical audits were conducted via the Institute for Healthcare Improvement (IHI) Global Trigger Tool (GTT) Framework (Griffin and Resar 2009). An audit of adverse events was performed via a modified GTT. Many of the original trigger tools for hospital adverse events from the GP Trigger Tool (Hibbert and Williams 2014) were not applicable to this VVED service. The triggers that were relevant and included were representation to the VVED, re-presentation to the ED, procedures in the ED, admission to the hospital, admission to the ICU, and death (Hibbert and Williams 2014). The IHI categorization for adverse events was used (Table 1). The outcomes of all re-presentations to both the VVED and the physical ED were independently investigated via electronic chart review by two medical practitioners (CM and JH). Two emergency physicians, one GP, and one pediatric emergency physician, reviewed and categorized adverse event cases independently (SM, JH, CM, LS). Contributing factors were identified, and a consensus was reached by the physicians who performed the chart review. Conflicts were resolved by consensus among all four physicians. Recommendations were developed and finalized by this group. Mortality data for the three-month period following the project were obtained from linkage to the Victorian Registry of Births Deaths and Marriages.

**Table 1:** Global trigger tool categories – Institute for Healthcare Improvement.

Category E: Temporary harm to the patient and required intervention ​ Category F: Temporary harm to the patient and required initial or prolonged hospitalization. ​ Category G: Permanent patient harm ​ Category H: Intervention required to sustain life ​ Category I: Patient died

Descriptive statistics were used to summarize the data, including sample characteristics and outcomes, via Microsoft Excel (Microsoft 2018). Means were used to describe the normally distributed variables of the sample. A chi-square test of independence was performed to examine the relationships between categorical data. The numbers, percentages and rates of participants who were seen and diverted from the ED were compared across the two study periods via the incidence rate ratio. This was performed via MedCalc for Windows, version 22.007 (Software M, n.d.).

### Ethics approval and consent to participate

This project was approved by St Vincent’s Human Research Ethics Committee (Project ID: 82094). A waiver of consent for the use of deidentified patient information was obtained from participants during the VVED registration process.

## Results

**Figure 2:**
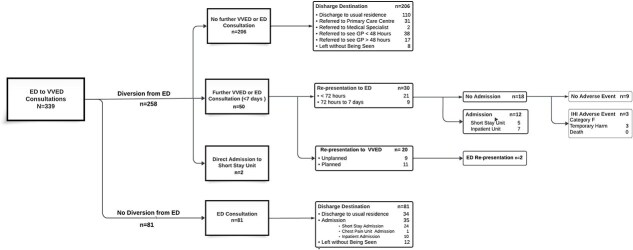
Outcomes of the emergency department to virtual (EDV) pathway.

A total of 47 022 patients presented to the ED during the study period, 13 981 of whom were Triage Category 4 or 5. A total of 339 referrals to the VVED occurred through the EDV pathway during the study period. The mean age of the patients was 32 years (range 0–91 years), and 52% were female. The patient outcomes of the consultations are shown in Fig. 2.

Of the 339 consultations with the VVED, 258 (76%) were diverted from the ED, whereas 81 (24%) were advised to visit the physical ED as originally planned. Following the original VVED presentation, 206 (61%) of the 258 diverted participants required no further consultation, 20 (6%) represented to the VVED, and 30 (9%) represented to the physical ED within a 7-day period. Two patients were directly admitted to a short-stay unit. No deaths were identified during the 3 months of follow-up. Fig. 2 provides an overview of the outcomes for patients who participated in the Emergency Department to Virtual Emergency Department (EDV) pathway. The outcomes used in the research were compulsory for the service, and no missing data occurred.

A high rate of subsequent physical ED representation occurred if the patient had previously attended the ED or been admitted in the previous seven days (n = 12, 41%) compared with those with no previous presentation or admission (n = 18, 5.8%). There was a significant association between prior ED attendance or hospital admission within the seven days preceding the EDV consultation and subsequent ED representation. (x^2^ (1, N = 339) 41.5, *P* < .001).

During the first study period of 9 weeks, only 35 patients were identified by the triage nurse, who were recommended to attend VVED (average of ~four consultations per week). Fourteen of these patients (40%) were diverted from the ED. During study period two, the subsequent 11-week period, a nurse navigator identified and directed patients to the EDV consultation. This resulted in 298 patient referrals to the VVED room (an average of 27 consultations per week). A total of 198 patients (66%) were diverted from the physical ED. A nurse navigator being employed was associated with higher rates of patient EDV consultations (IRR 7.3 (CI 5.1–10.3, *P* < .001)) and diversions from the ED (x^2^, 1, N-339) = 7.56, *P* < .01).

Following a review of all patients who re-presented to the ED within the seven days following VVED consultation, three adverse events were categorized as IHI Category F. This constituted 1% of all presentations. The clinical recommendations produced by the clinicians in the author group are detailed in Table 2.

**Table 2:** Clinical recommendations for the ED waiting room-to-virtual care (EDV) pathway.

(1) All observations need to be communicated to the VVED Clinician. Abnormal vital signs should be acknowledged, and it should be carefully considered whether these patients are suitable for VVED. (2) Robust measures to obtain past medical history are needed. This may include clinicians asking about history in multiple ways and improving state-wide medical health record integration. (3) Patients with an ED or inpatient attendance within the previous seven days should be identified and excluded from this pathway. (4) A previous related GP attendance or referral should have a senior clinician consultation, and a copy of the referral accessible to the VVED clinician. (5) Abdominal pain may not be suitable for EDV if there are no adequate options for a full assessment. (6) There were benefits in both the increased numbers of patients seen and the number of patients diverted by employing a nurse to facilitate the pathway. (7) Patients with non-English speaking backgrounds should either not be diverted to EDV or an interpreter should be mandated.

## Discussion

This unique study evaluated the occurrence of adverse events and the efficacy of a novel telehealth intervention and successfully diverted patients from the physical ED waiting room with few adverse outcomes. No deaths occurred following the diversion of patients to the VVED pathway, and 61% of patients were able to meet their secondary care needs entirely via virtual consultation. Nearly a quarter of patients identified as suitable for the EDV pathway were subsequently found to be unsuitable for VVED after consultation with a virtual medical practitioner. This is comparable to a previous study result of 22% (Osmanlliu et al. 2022). If patients had recently attended the ED or been admitted, they were less likely to be diverted from the ED after VVED consultation.

Dedicated nurse navigators improved the number and effectiveness of patients referred to the EDV pathway. The effectiveness of this approach may be derived from two mechanisms that influence effective nursing triage. First, the nurses were experienced in VVED use, and second, they were specifically trained for this service (Soola et al. 2022). The extended unified theory of acceptance and use of technology (UTAUT) model is a framework that can be applied to further understand the factors influencing the acceptance and use of technology (Cimperman et al. 2016). This approach is specifically tailored to different contexts, including telehealth (Cimperman et al. 2016). It has been shown that increased effort in using technology (Effort Expectancy), perceived usefulness (Performance Expectancy), and Perceived Security (e.g. data privacy) are the most important predictors of telehealth uptake (Cimperman et al. 2016). There is also a positive effect on the acceptance of use if an expert recommends a telehealth service (Cimperman et al. 2016). The VVED nurse navigator role strongly influences access to the telehealth service (Effort Expectancy), performance expectancy (explaining how it could be beneficial to patients) and the professional and expert recommendation of the nurse, in addition to the privacy of this intervention, as they were guided to the soundproof booth. The effectiveness of nurses in this role is supported by findings that improvements in the safety of triage nurses in the ED are associated with the level of experience and lack of interruptions (Fekonja et al. 2023). The opportunity costs involved in the staffing of ED clinicians involved in this pathway are important to consider in implementing this pathway. This is especially relevant given the current shortages in the ED workforce. The impacts from the small numbers of participants in this study are likely to be minor for both services involved. We are planning to embed the model statewide, and at this point, it is likely to have a measurable impact on outcomes such as wait times in addition to the costs and demands for the services*.*

The re-presentation rate of 6% of patients seen in the pathway is comparable to that of traditional physical EDs, which has been reported to range from 1.9% to 6.7% (Hutchinson et al. 2019). A study of ED diversion from the waiting room to a primary care facility revealed no difference in representation rates to the ED between those diverted from the ED and those not diverted (Doran et al. 2013). A single study of representations following ED telehealth reported the same rate of representation (Hsu et al. 2020). Likewise, pediatric studies on medical ED diversion via telehealth and face-to-face transmission from the waiting room revealed no difference in re-presentation (Osmanlliu et al. 2022; Ellbrant et al. 2015). The admission rate in our study was 40% for patients who represented, which is at the higher end of the range documented in a recent international literature review of face-to-face EDs (0.96%–40%) (Hutchinson et al. 2019). In the present study, admissions to an overnight bed in the ED (short-stay unit admission) were also included as admissions, which likely contributed to an increase in this number.

The adverse event rate for re-presentations in this study was 10%, which aligns with the range reported for face-to-face EDs of 5%–18% (Robinson and Lam 2013). Gasperini *et al.* reported an ~10% rate of adverse events following physician review of notes in face-to-face settings (Gasperini et al. 2023). A similar model to our intervention shows a comparable representation rate of 8%. In the study by Osmanlliu *et al.*, no adverse events were identified <72 h; however, only 27 patients were included. A large cohort study using a similar pathway to EDV revealed that the return time < 72 h and admission rate after these returns were not different between those seen in person and those seen by telehealth from the waiting room (Hsu et al. 2020). Patient satisfaction scores were similar between the two groups (Hsu et al. 2020). Further qualitative studies are needed to explore the experience of patients being diverted to a virtual service, given that a small number of patients refused the service when offered by a nurse navigator.

### Limitations

The follow-up of any participants in this study was only undertaken at a single local hospital. It is possible that they may have presented to alternative hospitals outside of the area. In similar studies of physical ED representations in Australia, this figure has been estimated to be low at 13% (Hutchinson et al. 2021). Further studies would benefit from the use of data linked to statewide hospital presentations to investigate this topic. Conversely, we have not examined whether introducing patients to the VVED in the ED waiting room resulted in future use of the VVED from home, potentially leading to future diversions. The use of alternative primary healthcare services was not documented in this study. However, a review of diversion from ED practices revealed no increase in unplanned primary care use for patients diverted from an ED (Kirkland et al. 2019). The current study may have been limited by any confounding interventions or impacts that occurred during the study period, such as a pandemic wave. There were no marked changes in the number of ED presentations during the study period. The current study was limited in that no comparison was undertaken. In addition, no assessment of patient acceptability was undertaken and would be a priority for future studies. Although previous studies have shown reduced wait times and good patient satisfaction, we acknowledge that any displeasure from having to continue to an ED consult after a VVED consult was not recorded. Prior to consultation, the patients consented to explain the process and the risk of having to continue to an ED consultation. In addition, during the process, they maintained their place in the waiting room queue.

The tool used to analyze adverse events has not been validated for virtual ED use. Further research into developing and validating suitable triggers for the IHI GTT is recommended in the telehealth setting. Although we included fewer triggers than the original GTT did, we analyzed every presentation and not a random sample to increase the sensitivity of this tool.

Further cost–benefit analysis is needed before hospitals can adopt this pathway, which is beyond the scope of this project and would influence any sustainability of this model.

## Conclusions

This study presents one of the earliest descriptions of the effectiveness and rate of adverse events following the diversion of patients from an ED waiting room to a virtual consultation. The role of a nurse navigator was identified as an important element in improving the effective diversion of low-acuity patients from the ED waiting room. We have proposed recommendations that may further enhance the utility, and efficiency of telehealth for this intervention, for which there has been little previous information.

## Supplementary Material

Supplementary_materials_oqag018

## Data Availability

Data will be available at the request to the authors.

## References

[ref1] Berchet C . Emergency Care Services : Trends, Drivers and Interventions to Manage the Demand. OECD Health Working Papers: Éditions OCDE, 2015, https://www.oecd.org/content/dam/oecd/en/publications/reports/2015/08/emergency-care-services_g17a26ec/5jrts344crns-en.pdf

[ref2] Bittencourt RJ, Stevanato AM, Braganca C et al. Interventions in overcrowding of emergency departments: an overview of systematic reviews. *Rev Saude Publica* 2020;54:66. 10.11606/s1518-8787.2020054002342 32638885 PMC7319499

[ref3] Cimperman M, Makovec Brenčič M, Trkman P. Analyzing older users' home telehealth services acceptance behavior-applying an extended UTAUT model. *Int J Med Inform* 2016;90:22–31. 10.1016/j.ijmedinf.2016.03.002 27103194

[ref4] Doran KM, Colucci AC, Hessler RA et al. An intervention connecting low-acuity emergency department patients with primary care: effect on future primary care linkage. *Ann Emerg Med* 2013;61:312–321.e7. 10.1016/j.annemergmed.2012.10.021 23261312

[ref5] Ellbrant J, Åkeson J, Åkeson PK. Pediatric emergency department management benefits from appropriate early redirection of nonurgent visits. *Pediatr Emerg Care* 2015;31:95–100. 10.1097/PEC.0000000000000348 25654674

[ref6] Fekonja Z, Kmetec S, Fekonja U et al. Factors contributing to patient safety during triage process in the emergency department: a systematic review. *J Clin Nurs* 2023;32:5461–77. 10.1111/jocn.16622 36653922

[ref7] Friedman J, Lame M, Clark S et al. Telemedicine medical screening evaluation expedites the initiation of emergency Care for Children. *Pediatr Emerg Care* 2021;37:e417–20. 10.1097/pec.0000000000002428 33848095

[ref8] Gasperini G, Bouazzi L, Sanchez A et al. Healthcare-associated adverse events and readmission to the emergency departments within seven days after a first consultation. *Front Public Health* 2023;11:1189939. 10.3389/fpubh.2023.1189939 37483920 PMC10359972

[ref9] Greenwald PW, Stern M, Clark S et al. A novel emergency department-based telemedicine program: how do older patients fare? *Telemed J E Health* 2019;25:966–72. 10.1089/tmj.2018.0162 30358524

[ref10] Griffin FA, Resar RK. IHI Global Trigger Tool for Measuring Adverse *Events (Second Edition)*. Cambridge, MA: IHI, 2009, https://www.ihi.org/library/white-papers/ihi-global-trigger-tool-measuring-adverse-events

[ref11] Grygorian A, Montano D, Shojaa M et al. Digital health interventions and patient safety in abdominal surgery: a systematic review and meta-analysis. *JAMA Netw Open* 2024;7:e248555. 10.1001/jamanetworkopen.2024.8555 PMC11053376 38669018

[ref12] Hibbert P, Williams H. The use of a global trigger tool to inform quality and safety in Australian general practice: a pilot study. *Aust Fam Physician* 2014;43:723–6. 25286432

[ref13] Houze-Cerfon C-H, Vaissié C, Gout L et al. Development and evaluation of a virtual research environment to improve quality of Care in Overcrowded Emergency Departments: observational study. *JMIR Serious Games* 2019;7:e13993. 10.2196/13993 31397292 PMC6705008

[ref14] Hsu H, Greenwald PW, Clark S et al. Telemedicine evaluations for low-acuity patients presenting to the emergency department: implications for safety and patient satisfaction. *Telemed J E Health* 2020;26:1010–5. 10.1089/tmj.2019.0193 31930952

[ref15] Hutchinson CL, McCloughen A, Curtis K. Incidence, characteristics and outcomes of patients who return to emergency departments. An integrative review. *Australas Emerg Care* 2019;22:47–68. 10.1016/j.auec.2018.12.003 30998872

[ref16] Hutchinson CL, Curtis K, McCloughen A et al. Identifying return visits to the emergency department: a multicenter study. *Australas Emerg Care* 2021;24:34–42. 10.1016/j.auec.2020.05.007 32593525

[ref17] Kirkland SW, Soleimani A, Rowe BH et al. A systematic review examining the impact of redirecting low-acuity patients seeking emergency department care: is the juice worth the squeeze? *Emerg Med J* 2019;36:97–106. 10.1136/emermed-2017-207045 30510034

[ref18] Microsoft . Microsoft Excel [Internet]. Redmond, WA, USA: Microsoft Corporation, 2018 [cited 2024 August 17th], Available from: https://office.microsoft.com/excel

[ref19] Morley C, Unwin M, Peterson GM et al. Emergency department crowding: a systematic review of causes, consequences and solutions. *PLoS One* 2018;13:e0203316. 10.1371/journal.pone.0203316 30161242 PMC6117060

[ref20] Osmanlliu EA-O, Gagnon I, Weber S et al. The waiting room assessment to virtual emergency department pathway: initiating video-based telemedicine in the pediatric emergency department. *J Telemed Telecare* 2022;28:452–7. 10.1177/1357633X211044038 34636683

[ref21] Peterson SM, Harbertson CA, Scheulen JJ et al. Trends and characterization of academic emergency department patient visits: a five-year review. *Acad Emerg Med* 2019;26:410–9. 10.1111/acem.13550 30102817

[ref22] Rademacher NJ, Cole G, Psoter KJ et al. Use of telemedicine to screen patients in the emergency department: matched cohort study evaluating efficiency and patient safety of telemedicine. *JMIR Med Inform* 2019;7:e11233. 10.2196/11233 31066698 PMC6530260

[ref23] Richardson DB . Increase in patient mortality at 10 days associated with emergency department overcrowding. *Med J Aust* 2006;184:213–6. 10.5694/j.1326-5377.2006.tb00204.x 16515430

[ref24] Robinson K, Lam B. Early emergency department representations. *Emerg Med Australas* 2013;25:140–6. 10.1111/1742-6723.12048 23560964

[ref25] Sharma R, Fleischut P, Barchi D. Telemedicine and its transformation of emergency care: a case study of one of the largest US integrated healthcare delivery systems. *Int J Emerg Med* 2017;10:21. 10.1186/s12245-017-0146-7 28685213 PMC5500596

[ref26] Shaver J . The state of telehealth before and after the COVID-19 pandemic. *Prim Care* 2022;49:517–30. 10.1016/j.pop.2022.04.002 36357058 PMC9035352

[ref27] Snoswell CL, Chelberg G, De Guzman KR et al. The clinical effectiveness of telehealth: a systematic review of meta-analyses from 2010 to 2019. *J Telemed Telecare* 2021;29:669–84. 10.1177/1357633X211022907 34184580

[ref28] Software M . MedCalc for Windows v22.007 Ostend. Belgium: Medcalc.

[ref29] Soola AH, Mehri S, Azizpour I. Evaluation of the factors affecting triage decision-making among emergency department nurses and emergency medical technicians in Iran: a study based on Benner's theory. *BMC Emerg Med* 2022;22:174. 10.1186/s12873-022-00729-y 36303127 PMC9613063

[ref30] Sprivulis PC, Da Silva JA, Jacobs IG et al. The association between hospital overcrowding and mortality among patients admitted via western Australian emergency departments. *Med J Aust* 2006;184:616–2. 10.5694/j.1326-5377.2006.tb00416.x 16515429

[ref31] Sun BC, Hsia RY, Weiss RE et al. Effect of emergency department crowding on outcomes of admitted patients. *Ann Emerg Med* 2013;61:605–611.e6. 10.1016/j.annemergmed.2012.10.026 23218508 PMC3690784

[ref32] Talevski J, Semciw AI, Boyd JH et al. From concept to reality: a comprehensive exploration into the development and evolution of a virtual emergency department. *J Am Coll Emerg Physicians Open* 2024;5:e13231. 10.1002/emp2.13231 39056087 PMC11269764

[ref33] Tolia V, Castillo E, Guss D. EDTITRATE (emergency department telemedicine initiative to rapidly accommodate in times of emergency). *J Telemed Telecare* 2016;23:484–8. 10.1177/1357633X16648535 27279469

[ref34] Youssef E, Benabbas R, Choe B et al. Interventions to improve emergency department throughput and care delivery indicators: a systematic review and meta-analysis. *Acad Emerg Med* 2024;31:789–804. 10.1111/acem.14946 38826092

