# Supplementary material for: The effectiveness of the diversion of patients from an emergency department waiting room to a virtual medical consultation
Source: Oxf Open Digit Health. 2026 Jul 11;4:oqag018. doi: 10.1093/oodh/oqag018 (PMC13386168; doi:10.1093/oodh/oqag018)

Supplementary Material 2. Photo of room adjacent to waiting room in which EDV consult occurred.

Yellow square indicates VLN nurse position during consult.


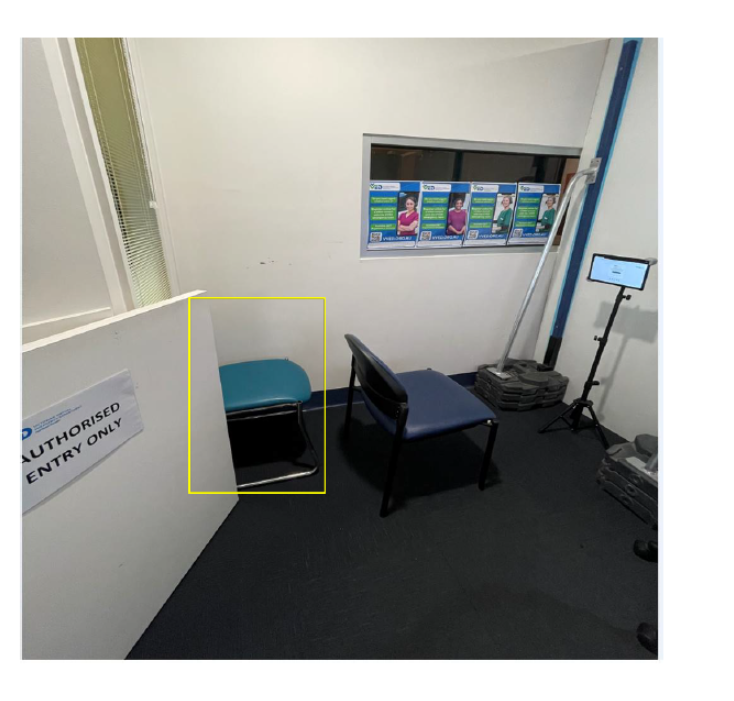

Supplement: Supplementary_materials_oqag018 [file supplementary_materials_oqag018.zip › EDV2804FinalSupplementary Material 2.docx]
