# Supplementary material for: The effectiveness of the diversion of patients from an emergency department waiting room to a virtual medical consultation
Source: Oxf Open Digit Health. 2026 Jul 11;4:oqag018. doi: 10.1093/oodh/oqag018 (PMC13386168; doi:10.1093/oodh/oqag018)
Supplement: Supplementary_materials_oqag018 [file supplementary_materials_oqag018.zip › FinalSupplementaryMaterial1.VLN NH ED ONLY - Inclusion and Exclusion Criteria for Patient Referrals to VVED.pdf]

Supplementary Material 1.

Inclusion and Exclusion Criteria for Patient Referrals to VVED

Appropriate Patients	Requires Consideration Prior To Referral	Inappropriate Patients
• Body aches/pains<br>• Constipation<br>• Coryzal symptoms<br>• COVID-19<br>• Cough<br>• Diarrhoea<br>• Eye discharge<br>• Early dehydration<br>• Earache<br>• Epistaxis (isolated & resolved, not anticoagulated)<br>• Fever in paediatrics (> 3 months old)<br>• Head injury (mild)<br>• Haemorrhoids<br>• Insect bites<br>• Mastitis<br>• Nausea/vomiting<br>• PR/PV bleeding (mild) inc. early pregnancy with confirmed intrauterine pregnancy<br>• Rash<br>• Shortness of breath (mild)<br>• Sore throat<br>• Urinary symptoms	• Abdominal pain <60 years<br>• Back pain <60 years<br>• Chest pain (low risk)<br>• Dizziness <60 years<br>• Foreign body of nose, eyes or ears<br>• Headache<br>• Hypertension<br>• Lacerations/abrasions<br>• Leg swelling<br>• Limb injuries<br>• Shortness of breath (moderate)<br><br>***Use clinical judgement and/or seek advice regarding these patients (VVED ANUM on teams)**	• Alcohol or drug effected patients<br>• Altered conscious state (GCS <15)<br>• Arrythmias<br>• Chest pain >35 years (moderate/high risk)<br>• High risk mental health patients including self- harm or overdose<br>• Refilling scripts<br>• Medical certificates<br>• Severe pain or patient likely to require opiates, benzodiazapines or other scheduled<br>• Severe respiratory illness (eg. unable to speak in sentences)<br>• Stroke like symptoms<br>• Trauma including obviously deformed limbs<br>• Under 3 months of age with fever<br><br>**VVED is <u>not</u> to be used by patients who do not have a device that has video and audio capabilities and are unable to use telehealth technology independently**
